# High-Fat, High-Calorie Diet Enhances Mammary Carcinogenesis and Local Inflammation in MMTV-PyMT Mouse Model of Breast Cancer

**DOI:** 10.3390/cancers7030828

**Published:** 2015-06-26

**Authors:** Sarah Cowen, Sarah L. McLaughlin, Gerald Hobbs, James Coad, Karen H. Martin, I. Mark Olfert, Linda Vona-Davis

**Affiliations:** 1 Department of Surgery, West Virginia University Health Sciences Center, Morgantown, WV 26506, USA; E-Mail: scowen@miis.edu; 2 Mary Babb Randolph Cancer Center, West Virginia University Health Sciences Center, Morgantown, WV 26506, USA; E-Mails: smclaughlin@hsc.wvu.edu (S.L.M.); ghobbs@stat.wvu.edu (G.H.); kamartin@hsc.wvu.edu (K.H.M.); imolfert@hsc.wvu.edu (I.M.O.); 3 Department of Statistics, West Virginia University, Morgantown, WV 26506, USA; 4 Department of Pathology, West Virginia University Health Sciences Center, Morgantown, WV 26506, USA; E-Mail: jcoad@hsc.wvu.edu; 5 Department of Neurobiology and Anatomy, West Virginia University Health Sciences Center, Morgantown, WV 26506, USA; 6 Department of Human Performance and Exercise Physiology, West Virginia University Health Sciences Center, Morgantown, WV 26506, USA

**Keywords:** obesity, high-fat diet, inflammation, angiogenesis, tumor progression

## Abstract

Epidemiological studies provide strong evidence that obesity and the associated adipose tissue inflammation are risk factors for breast cancer; however, the molecular mechanisms are poorly understood. We evaluated the effect of a high-fat/high-calorie diet on mammary carcinogenesis in the immunocompetent MMTV-PyMT murine model. Four-week old female mice (20/group) were randomized to receive either a high-fat (HF; 60% kcal as fat) or a low-fat (LF; 16% kcal) diet for eight weeks. Body weights were determined, and tumor volumes measured by ultrasound, each week. At necropsy, the tumors and abdominal visceral fat were weighed and plasma collected. The primary mammary tumors, adjacent mammary fat, and lungs were preserved for histological and immunohistochemical examination and quantification of infiltrating macrophages, crown-like structure (CLS) formation, and microvessel density. The body weight gains, visceral fat weights, the primary mammary tumor growth rates and terminal weights, were all significantly greater in the HF-fed mice. Adipose tissue inflammation in the HF group was indicated by hepatic steatosis, pronounced macrophage infiltration and CLS formation, and elevations in plasma monocyte chemoattractant protein-1 (MCP-1), leptin and proinflammatory cytokine concentrations. HF intake was also associated with higher tumor-associated microvascular density and the proangiogenic factor MCP-1. This study provides preclinical evidence in a spontaneous model of breast cancer that mammary adipose tissue inflammation induced by diet, enhances the recruitment of macrophages and increases tumor vascular density suggesting a role for obesity in creating a microenvironment favorable for angiogenesis in the progression of breast cancer.

## 1. Introduction

Epidemiological studies have shown that obesity is a risk factor for breast cancer in postmenopausal women, when the tumors are frequently estrogen receptor (ER)-positive and a major mechanism involves elevated aromatase activity and estrogen production in adipose tissue stromal cells [1,2,3]. In general, obesity was found not to be a positive risk factor for premenopausal breast cancer and, indeed to exert a protective effect, at least in younger women [2]. Obesity is also associated with more advanced disease at the time of an initial breast cancer diagnosis and with a poor prognosis, but here the disease is not influenced by menopausal status [4].

Adipose tissue inflammation is causally related to obesity-related metabolic disorders, such as insulin resistance and type 2 diabetes, dyslipidemias and atherosclerotic heart disease, and, perhaps, some cancers including carcinoma of the breast [5,6,7,8]. Among the contributors to the biochemical and molecular mechanisms are the proinflammatory cytokines such as tumor necrosis factor-α (TNF-α) and interleukin-6 (IL-6), and the chemokine monocyte chemoattractant protein-1 (MCP-1) which are produced by the macrophages that infiltrate the adipose tissue, and do so in increased numbers in obesity [9,10], the preadipocytes, and mature adipocytes [11,12]. Histologically, this inflammation has been characterized by “crown-like structures” (CLS) which are formed by aggregation of the infiltrating macrophages around individual adipocytes with resulting cell necrosis and formation of a syncytium of lipid-containing giant multinucleated cells and were shown to be present in increased numbers in obese mice and humans [13,14].

Neovascularization occurs in adipose tissue to meet the demands of an expanding body fat mass, a process which is regulated by vascular endothelial growth factor (VEGF) and other protein angiogenic mediators, such as leptin [15] and MCP-1 [16,17] secreted by pre-adipocytes and macrophages. Dietary-induced obesity in mice was prevented by treatment with a pharmacological inhibitor of angiogenesis [18]. Angiogenesis is induced by inflammation [19] and this relationship may be an essential component of the altered microenvironment that favors breast cancer growth and metastasis [15].

The objective of the study reported here was to use an animal model to investigate the influence of obesity and inflammation produced by feeding a high-fat, high-calorie, diet on the entire process of breast carcinogenesis from the stages of initiation and early proliferation at the primary site to the formation of systemic metastases, and on tumor-associated angiogenesis. Chemically-induced rat mammary tumors are influenced by the nature and level of dietary fat consumption, but they do not metastasize, or do so only with low frequency [20,21], and although both the growth and metastasis of ER-negative human breast cancer cell lines in athymic nude mice are stimulated by high omega-6 fatty acid intake [22] the model by-passes the early steps in cancer development. Therefore, in this pilot study we used the transgenic polyoma middle T oncoprotein (PyMT) mammary tumor-bearing mouse, whose tumors progress through all the stages from adenoma formation, low-grade carcinoma, and high-grade cancer with high metastatic potential [23]. Gordon *et al.* [24,25] had shown previously that in this model cancer growth and metastasis are enhanced by cosegregation with obesity quantitative trait loci (QTL). Mice fed a high-fat (45%–60%) diet develop obesity, abnormal glucose utilization, and insulin resistance: features of chronic adipose tissue inflammation and type 2 diabetes [26,27,28].

## 2. Experimental Section

### 2.1. Animals

Female MMTV-polyoma middle T antigen (PyMT) transgenic mice on an FVB background (double transgenic; FVB/N-Tg [MMTV-PyVT] 634 Mul∙J^−1^), were purchased from Jackson Laboratories (Bar Harbor, ME, USA). They were approximately 4 weeks of age at delivery and were housed in a room with controlled temperature (22–24 °C) and relative humidity (50%–60%) under a 12 h:12 h light-dark cycle. The FVB/N strain is relatively resistant to the development of obesity when fed a HF diet compared with some other strains such as the C57BL/BJ mouse [29]. These animal studies were approved by the West Virginia University Research Compliance Office and carried out in compliance with regulations established by the West Virginia University Institutional Animal Care and Use Committee. Animal care and handling were performed in accordance with the US Public Health Service Animal Welfare Act and conformed to the principles and procedures dictated by the highest standards of humane animal care.

### 2.2. Experimental Diets

The high-fat (HF) and low-fat control (LF) pelleted diets were purchased from BioServ Inc. (Frenchtown, NJ, USA). The HF diet (certified diet F3284) contained 36% fat in the form of lard (60% total kcal) and provided 5.49 kcal∙g^−1^. The protein source was casein and dl-methionine (20.5%), the carbohydrate source was maltodextrin and sucrose (35.7%) and the minerals were a mix. The fat contained 40% saturated, 48% mono- and 12% polyunsaturated fatty acids. The LF diet (certified diet F4031) contained 7% fat from lard (16% total kcal) and provided 3.93 kcal∙g^−1^. The protein source was casein and dl-methionine (20.5%), the carbohydrate source was cornstarch, sucrose and maltodextrin (35.7%) and the minerals were a mix identical to the HF diet. These diets have been used extensively for obesity studies in mice [26,28,30], including the modeling of obesity and mammary adipose tissue inflammation [14]. Food and demineralized drinking water were supplied *ad libitum.*

### 2.3. Experimental Procedure

The mice, 20 animals per group were assigned randomly to receive the HF or LF diet, commencing at age 4 weeks and continuing for 8 weeks. Body weights and tumor volumes were determined weekly. Timepoints were selected at 4 and 8 weeks post diet consumption to measure histologic changes, *n* = 9−10 mice/diet/timepoint. The duration of the experimental period was limited by size of the primary tumors and the occurrence of skin ulceration. At termination, blood was collected and the plasma stored at −80 °C prior to cytokine analyses. Full necropsies were performed at which the primary tumors and the maximal amount of visceral abdominal fat possible were excised, weighed, and, together with the liver and lungs, fixed with formalin and embedded in paraffin for later histological examination. Histopathology and immunohistochemistry in the Pathology Laboratory for Translational Medicine (Morgantown, WV, USA).

### 2.4. Primary Tumor Volume Measurements

These were made by ultrasound using a VisualSonics Vevo 2100 *in vivo* high-resolution microimaging system (VisualSonics, Toronto, ON, Canada), which provided 3 dimensional images of the right and left 4th and 5th (inguinal) mammary glands and tumor mass volumes (mm^3^). The method permits early detection and more accurate determination of size at an early stage of solid tumor growth than reliance on caliper measurements. Briefly, the mice were anesthetized with an isoflurane-oxygen mixture, and a transducer placed over the mammary gland which was scanned to a depth of 15 mm with an axial resolution of 40 μm. Tumor volumes were calculated using the integrated Vevo 2100 software (version 1.6.0, VisualSonics, Toronto, ON, Canada).

### 2.5. Lung Metastases

The entire 5 μm, hematoxylin and eosin-stained, organ cross-section was scanned by bright-field light microscopy in adjacent but non-overlapping fields at 100 × magnification. For each organ, the total number of micrometastases was counted and the diameter of the largest in the plane of the section was measured with a calibrated ocular micrometer equipped with an Olympus Calibration Slide 1x3x.110 (Klarmann Rulings, Litchfield, NH, USA). The total area of each organ cross-section was determined using calibrated digital photomicrograph imaging with Infinity Analyze and Capture software (version 6.5.1, Lumenera Corporation, Ottawa, ON, Canada), and the number of metastases/square mm calculated from the total micrometastases and organ area.

### 2.6. Hepatic Steatosis

The presence of hepatocellular macrovesicular and microvesicular steatosis was determined in hematoxylin and eosin-stained 5 μm tissue sections with scanning of the entire organ cross section by bright-field light microscopy at 40×; microvesicular steatosis was confirmed with 200 × magnification. Steatosis was graded according to the percentage of hepatocytes showing intracellular lipid accumulation: trace, 5%–25%; mild, 25%–50%; moderate, 50%–75%; marked, 75%–100%.

### 2.7. Infiltrating Macrophages and Microvessel Densities

Tissues were deparaffinized and rehydrated prior to antigen retrieval, and 4 μm sections stained by a standard immunohistochemical technique using a rat anti mouse CD68 for the macrophage cell marker and anti-CD31 primary rabbit polyclonal antibody for vascular endothelium (abCam, Cambridge, MA, USA). The anti-CD68 was diluted 1:100 and the anti-CD31 1:500 in Dako Background Reducing Diluent, with incubation times of 8 h and 3 h, respectively. Hematoxylin provided the background staining. Mouse splenic tissue was used as a positive control for the CD68 binding reaction.

For image acquisition, entire tissue sections containing tumors were scanned using the MBF Virtual Slice extension module of Stereo Investigator (MBF Bioscience, Williston, VT, USA) on an Olympus AX70 Provis microscope (Center Valley, PA, USA) using a 20 × 0.70 UplanApo objective and an Optronics MicroFire color CCD camera (Goleta, CA, USA).

Macrophages were counted manually in 10 random 313 × 313 μm fields. To determine microvessel densities, CD31 staining (DAB) in 10 random fields was separated from the RGB image using the ImageJ color deconvolution function with the preset H-DAB settings. Images were threshold highlighted to select areas with the highest intensity of brown color. The area of the thresholded staining compared to the entire field was used to calculate vascular density.

### 2.8. Crown-Like Structures

Adipose tissue sections adjacent to the tumor beds were selected for quantitation of CLS. Using an RGB image with a set scale of 1600 pixels = 589 μm, 313 µm × 313 μm squares were outlined for each section, and enclosed CLSs identified as clusters of macrophages that had infiltrated the adipose tissue and formed ring-like structures, and manually counted in 10 random fields.

### 2.9. Plasma Cytokines and Tissue MCP-1

BD™ cytometric bead array (CBA) Mouse Inflammation Kit (BD Biosciences, San Jose, CA, USA) was used for the cytokine assays. The technique, which is a capture multi-analyte immunoassay, uses amplified fluorescence with flow cytometry to determine TNF-α, IL-6, IL-10 and MCP-1. The limit of detection for MCP-1 is 52.7 pg∙mL^−1^, which is approximately 5- to 10-fold that of the other analytes, but still satisfactory for plasma assays. Interassay CVs at the relevant ranges are 5%–10%, and intra-assay CVs 2%–4%. A Quantikine^®^ ELISA kit from R&D Systems, Inc. (Minneapolis, MN, USA) was used to measure plasma leptin. The limit of detection was 22.0 pg∙mL^−1^ and the intra-assay CV was less than 5%. MCP-1 protein expression in tissue lysates (tumor and adipose) were measured using the Meso Scale Discovery Multi-Array electrochemiluminescence detection system (Meso Scale Discover, Gaithersburg, MD, USA). Lysates were normalized for protein content. The assay has a dynamic range of 1–10,000 pg∙mL^−1^. The intensity of emitted light was read on a MSD Sector Imager 2400 instrument (Meso Scale Diagnostics, LLC, Rockville, MD, USA).

### 2.10. Statistical Analyses

Repeated measures analysis of variance was used to assess differences for quantitative responses. The distribution of hepatic steatosis was analyzed using the Mantel-Haenszel Chi-square test for trend, using standardized mid ranks. All data are reported as the mean ± SEM. All tests were 2-sided, *p* < 0.05 being regarded as significant. Analyses were conducted with JMP Version 10.0 Software (SAS Institute, Cary, NC, USA).

## 3. Results

### 3.1. High-Fat, High Calorie Diet Increases Adiposity, Weight Gain and Hepatic Steatosis

**Figure 1:**
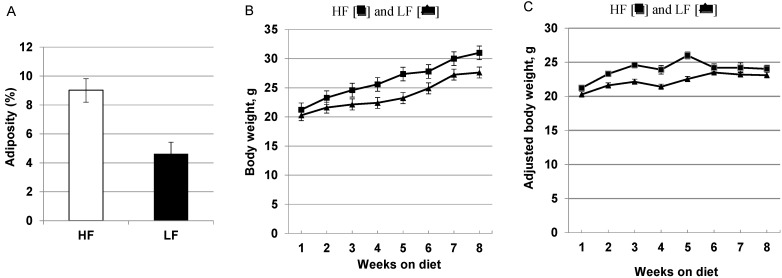
Body composition and weight gain in MMTV-PyMT mice following short-term (8 weeks) dietary intervention with high-fat (HF) and low-fat (LF) diets, *n* = 20/group. (**A**) Visceral adipose tissue as % body weight for the HF and LF-fed groups (*p =* 0.002); (**B**) Body weight gain over the 8 week observation period for HF [■] and LF [▲] fed mice. The greater weight gain in the HF group was statistically significant (*p* = 0.005). (**C**) Body weight gain adjusted for tumor weight over the 8 week diet period for HF [■] and LF [▲] fed mice was significant (*p* < 0.05).

The terminal mean abdominal visceral fat weight was 1.69 ± 0.30 g for the HF and 0.81 ± 0.11 g for the LF-fed mice; expressed as a percentage of the body weight, the acquisition of adiposity was significantly greater in the mice fed the HF diet (Figure 1A; *p =* 0.002). The body weight gain was greater for the mice fed the HF (60% total kcal) diet than for mice fed the LF (16% total kcal) diet (Figure 1B; *p* = 0.005). After 8 weeks, the mean body weight of the mice fed the HF diet was 31.00 ± 1.22 g and for the LF diet-fed mice it was 27.63 ± 2.25 g. The mice fed a HF diet gained more weight than the LF mice, even after adjustment for tumor burden (Figure 1C; *p* < 0.050). The average weight gain over the period of 5 weeks was 5.47 ± 0.47 g compared to 3.68 ± 0.48 g for the high and low-fat diets, respectively. 

At the end of the experiment, hepatic steatosis was assessed in 14 mice from the HF and 14 mice from the LF dietary groups, to provide an independent index of obesity-related metabolic disturbance. As shown in Table 1, a mild-moderate degree of steatosis was present in all of the animals fed the HF, but only 3 (21.4%) of those fed the LF diet (*p* < 0.001).

**Table 1:** Liver steatosis in tumor-bearing mice fed high-fat (HF) and low-fat (LF) diets at 4 and 8 weeks.

4 weeks		**Absent**		**Trace**		**Mild**		**Moderate**		*p*-value *
		***n***	**%**	***n***	**%**	***n***	**%**	***n***	**%**	
	LF	1	16.67	2	33.33	3	50.00	0	0.00	0.435
	HF	1	16.67	4	66.67	1	16.67	0	0.00	
8 weeks		**Absent**		**Trace**		**Mild**		**Moderate**		*p*-value *
		***n***	**%**	***n***	**%**	***n***	**%**	***n***	**%**	
	LF	3	21.43	8	57.14	3	21.43	0	0.00	<0.0001
	HF	0	0.00	0	0.00	11	78.57	3	21.43	

* Extract *p*-value from Mantel-Haenszel Chi-Square test for trend (using standardized mid ranks).

### 3.2. Chronic Consumption of a High-Fat Diet Significantly Increases Primary Mammary Tumor Growth without Effects on Latency in Female MMTV-PyMT Mice

**Figure 2:**
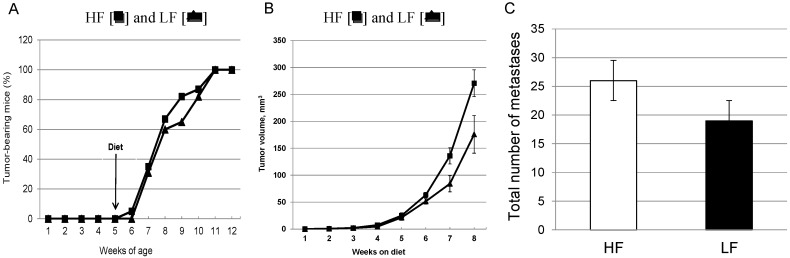
Period of tumor latency and primary tumor volume as measured by ultrasound. (**A**) Lack of effect of high-fat HF [■] and low-fat LF [▲] diets on latency for mammary tumor development, *n* = 20/group; (**B**) Primary tumor as measured in the 4th and 5th mammary for each mouse over the 8 week observation period for HF [■] and LF [▲] fed mice, *n* = 20 HF and *n* = 19 LF mice. The overall greater growth rate for the HF group was statistically significant (*p =* 0.0495). (**C**) Lack of effect on the total number of lung metastases over the 8 week observation period for LF and HF diet, *n* = 18/group.

Latency, as defined by the time to the detection of the first mammary tumor, ranged from 6 to 11 weeks, but overall was no different in mice fed the HF or LF diets (Figure 2A). At the end of the 8-week experimental period, all of the mice had histologically confirmed primary mammary carcinomas. The aggregate volumes of tumors growing in the left and right-sided 4th and 5th mammary fat pads are shown in Figure 2B. After a 5-week lag period, the increase in tumor volume in mice fed the HF diet was significantly greater than that in the LF diet-fed mice (*p* = 0.049). The mean tumor burden per mouse determined at necropsy were 3.70 ± 0.71g for the HF, and 2.62 ± 0.71g for the LF groups (*p* = 0.293).

Histological examination showed that all of the mice, in each of the two dietary groups, had some degree of lung involvement. The metastatic burden to the lung was greater in the HF-fed group for the total number of metastases (Figure 2C; HF, 26 ± 10; LF, 19 ± 5; *p* = 0.779), the number of metastases∙mm^2^ (HF, 0.64 ± 0.05 mm^2^; LF, 0.26 ± 0.09 mm^2^; *p* = 0.504), and the greatest tumor dimension (HF, 0.60 ± 0.25 mm; LF, 0.35 ± 0.06 mm; *p* = 0.410), but none reached statistical significance.

### 3.3. Diet-Induced Obesity Increases Tumor-Associated Macrophage Infiltration, Crown-Like Structures and Microvessel Density

**Figure 3:**
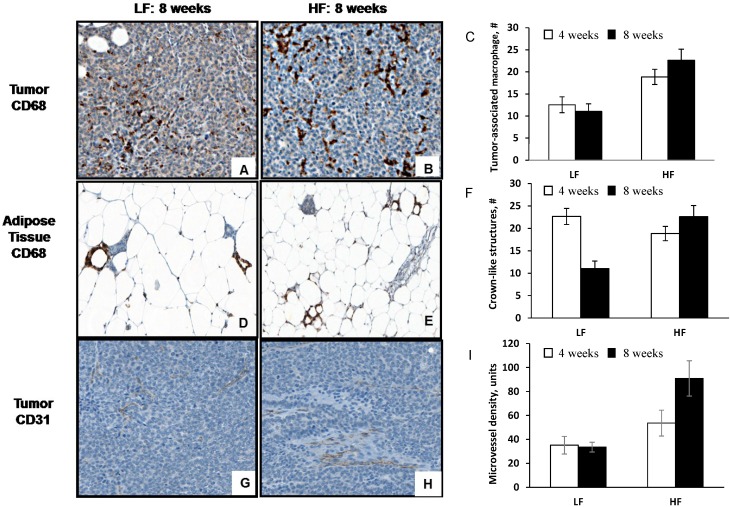
Macrophage infiltration into peritumoral adipose tissue and adjacent tumor mass. The macrophages were identified by their immunoreactivity with an anti-CD68 antibody and counted as described in the text for HF and LF fed mice. (**A**/**B**) Infiltration was significantly greater in the tumors from the HF-fed mice, *n* = 20/group compared to LF, *n* = 19/group (*p* = 0.0012); (**C**) Quantitation of tumor-associated macrophages at 4 and 8 weeks after diet consumption; (**D**/**E**) Representative peritumoral adipose tissues from HF and LF-fed mice showing crown-like structures (CLS) formed by the localization of macrophages around a single adipocyte. Their presence was increased in tissues from mice fed a HF diet; (**F**) Quantitation of crown-like structures at 4 and 8 weeks after diet consumption, *n* = 9–10 mice/diet/timepoint; (**G**/**H**) Tumor-associated angiogenesis and inflammatory changes in mice fed HF or LF diets. Microvessel density is indicated by endothelial cell immune-reactivity with anti-CD31 antibody and was significantly greater in tissues from HF-fed mice (*p* = 0.0002); (**I**), quantitation of microvessel density at 4 and 8 weeks after diet consumption, *n* = 9–10 mice/diet/timepoint.

There was extensive macrophage infiltration of the mammary tumor beds and the adjacent adipose tissue at the final endpoint of 8 weeks (Figure 3A,B). Tumor-associated macrophages, were significantly greater in the mice fed the HF diet compared to the control LF diet when measured at 4 and 8 weeks (Figure 3C). By 8 weeks, the average number of macrophage were 20.7 ± 1.3 and 16.8 ± 1.5 cells/tissue area, HF and LF, respectively; (*p* = 0.001). Macrophage that had invaded the adjacent adipose tissue and formed crown-like structures (Figure 3D,E). Crown-like structures were counted in the peritumoral adipose tissues at two time points; the difference in the number present for the two dietary groups just failed to achieve statistical significance (HF, 15.0 ± 0.1; LF, 9.0 ± 0.1; Figure 3F; *p* = 0.054). 

The microvessels formed in response to angiogenic stimulation were concentrated in the peritumoral adipose tissue and adjacent peripheral zones of the tumor mass (Figure 3G,H). Within the ten regions of interest randomly selected, the % area stained for CD31 showed that microvessel density was significantly greater in mice fed the HF compared with the LF diet for 8 weeks (HF, 88 ± 4; LF 32 ± 4; Figure 3I; *p* < 0.001).

### 3.4. Diet-Induced Obesity and Inflammation Significantly Increases Plasma Cytokines and Mammary Tumor Mcp-1 Production

There was a trend for higher plasma cytokine levels to be higher in the HF-fed mice (Table 2), which for IL-6 approached statistical significance (*p* = 0.059). Plasma MCP-1 and leptin concentrations were both elevated in the HF dietary groups (*p* = 0.002 and < 0.050, respectively).

**Table 2:** Plasma cytokines, MCP-1 and leptin concentrations in mice fed high-fat (HF) and low-fat (LF) diets.

Plasma Protein	Units	HF	LF	*p-*value
TNF-α	pg/mL	11.8 ± 2.0	7.5 ± 1.0	0.246
IL-6	pg/mL	3.0 ± 0.1	1.5 ± 0.2	0.059
IL-10	pg/mL	20.1 ± 4.0	14.0 ± 0.2	0.410
MCP-1	pg/mL	38.0 ± 0.3	22.0 ± 0.2	0.002
Leptin	ng/mL	8.3 ± 1.4	2.1 ± 0.5	<0.050

**Figure 4:**
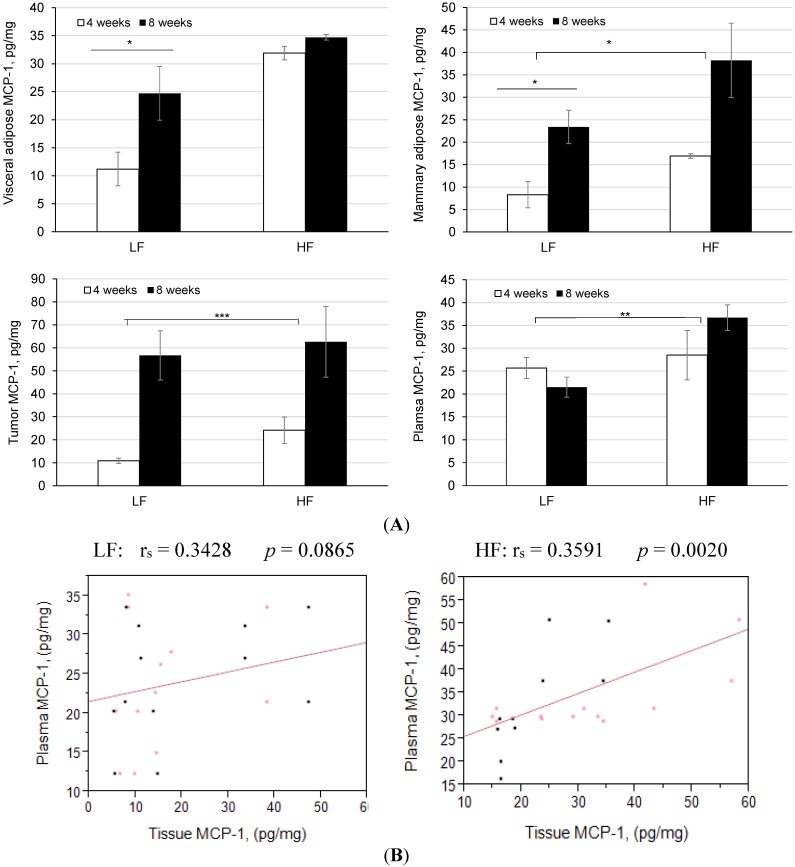
Tissue and plasma levels of MCP-1 and the effects of feeding a high-fat (HF) diet in tumor-bearing MMTV-PyMT mice. (**A**) Visceral adipose MCP-1 concentrations were higher at 4 weeks in the mice on HF diet (* *p* < 0.05); mammary adipose MCP-1 concentrations were higher at 4 and 8 weeks in mice fed HF diet (* *p* < 0.05); tumor tissue MCP-1 levels were significantly increased in both dietary groups (*** *p* < 0.001); plasma MCP-1 concentrations were significantly higher in the LF/HF groups (** *p* < 0.01). Data are depicted as mean ± S.E.M.; (B) MCP-1 expression in tissues (adipose, tumor) significantly correlates with plasma levels in mice fed HF diet (r_s_ = 0.3591, *p* = 0.002).

To determine if plasma MCP-1 concentrations reflect the adipose tissue and/or tumor levels, we measured the relative MCP-1 protein expression in response to a HF diet. The visceral adipose tissue MCP-1 concentrations were significantly higher in the HF compared with the LF (control) group at 4 weeks (Figure 4A; *p* < 0.050), a difference that was partially obscured at 8 weeks by increases in the LF fed mice (11.2 ± 3.0 *vs*. 24.7 ± 4.8 pg∙mL^−1^ protein). The MCP-1 levels in mammary adipose tissue were also higher in the HF-fed mice at 4 weeks, but in addition both dietary groups showed an increase at 8 weeks (all *p* < 0.050); again the higher mean MCP-1 level in the HF- compared with the LF-fed mice (38.2 ± 8.3 and 23.4 ± 3.7 pg∙mL^−1^ protein, respectively) did not achieve statistical significance. The tumor tissues showed a particularly pronounced increase in MCP-1 with time in both dietary groups (*p* < 0.001); there was a trend towards higher levels in the HF group after 8 weeks, but with high variance. Plasma MCP-1 concentrations in the LF-fed mice were similar at 4 and 8 weeks, whereas the HF group showed a time-related increase and were also significantly higher than those of the LF group after 8 weeks (*p* < 0.010).

To determine if there was a positive relationship between MCP-1 in the tissues and what circulates in the plasma of mice consuming the HF diet, we calculated correlation coefficients for plasma MCP-1 values with levels found in visceral adipose, mammary adipose, and tumor. Several weak correlations between plasma MCP-1 and tissue MCP-1 were found in body fat and mammary fat, with the exception of tumor MCP-1 which showed a positive correlation with plasma levels in mice fed the HF diet (r_s_ = 0.6675, *p* = 0.035; Figure 4B). The production of MCP-1 when combined for all tissues, correlated significantly with plasma MCP-1 for the HF mice (r_s_ = 0.3591; *p* = 0.002).

## 4. Discussion

In the present study, the MMTV-PyMT transgenic model of spontaneous mammary cancer was used to examine the interactions between a pre-existing and genetically determined enhanced breast cancer risk, adiposity and the associated adipose tissue inflammation resulting from a high dietary fat intake, and their relationship to angiogenesis. The HF diet promoted tumor volume as assessed by the growth rate of the primary tumors once they were detectable by ultrasound, and the severity of the metastatic burden in the lungs, effects that were consistent with the observed difference in tumor-related angiogenesis as indicated by microvessel densities in the two dietary groups. Moreover, the presence of inflammatory biomarkers and hepatic steatosis were common in mice fed the HF diet. By the end the study, the tumor burdens in the HF diet mice were greater than 10% of baseline body weight, indicating a somewhat lower body condition.

Obesity, low-grade inflammation, hepatic steatosis, and nonalcoholic steatohepatitis occur in association with type 2 diabetes, and insulin resistance and other components of the metabolic syndrome [31] which in epidemiological studies have been causally related to breast cancer [32]. Moreover, a state of abdominal tissue inflammation with the same metabolic complications as those attributed to obesity, but without an elevated body mass index, a condition of “metabolically obese-normal weight” [33], may also be responsible for an increased breast cancer risk.

In our study, the expansion of the adipose tissue mass without visually overt obesity in the HF-fed mice and the associated acceleration of MMTV-PyMT tumor progression were accompanied by the concentration of macrophages and CLS formation in the mammary adipose tissue, pathological changes that are consistent with inflammation. Similar abnormalities were described by Subbaramaiah *et al.* [14] in female mice fed the same HF diet, together with higher levels of TNF-α and IL-1β compared with LF-fed controls in the macrophage-rich stromal-vascular cellular fraction isolated from mammary glands, changes that were particularly prominent in mice with greater body weight gains due to a combination of a HF diet and ovariectomy. In both intact mice of the obesity-prone C57BL/6J strain [14] and the obesity QTL/MMTV-PyMT model [24], feeding a HF diet for 7 to 10 weeks produced animals with body weights that were approximately 5g higher than those fed a LF control diet. In contrast, in the present study the difference after 8 weeks was only 3.4 g, which included the contribution made by the higher mammary tumor weights. It is interesting to note that the mice did not become grossly obese compared to the mice on regular chow suggesting that some of the critical changes affecting tumor growth happen early in diet-induced metabolic obesity. This mouse model is an important one because tumors develop which more closely mimic human progression. Our study is also consistent with the concept of “metabolic obesity” described by Wildman *et al.* [33]. Morris *et al.* [34] not only demonstrated the presence of inflammation, with CLS formation and increased proinflammatory cytokine production, in the breast adipose tissue of obese women, but also noted that elevated aromatase expression and CLS number were more closely related to the severity of the inflammation than the level of excess adiposity as indicated by the body mass index.

The proinflammatory cytokines and chemokines secreted by macrophages and adipose cells, including TNF-α, IL-6, MCP-1 and leptin, can directly stimulate breast cancer cell proliferation and invasion. It is the M1 macrophage phenotype which is associated with obesity and adipose tissue inflammation, is responsible for TNF-α and IL-6 production, and may predominate during the early stages of tumorigenesis. At a later timepoint, a phenotypic shift may occur and be responsible for the accumulation of anti-inflammatory, but proangiogenic M2 macrophages, which secrete IL-10, within the tumor mass (reviewed in [8]). Further studies are warranted to investigate these relationships in the MMTV-PyMT model.

Subbaramaiah *et al.* [14] also found that aromatase activity was elevated in the mammary and visceral abdominal fat of the HF-fed mice. This enzyme, which is responsible for the extraglandular synthesis of estrogens from C19 steroids, is increased in the presence of adipose tissue inflammation, being inducible by TNF-α, IL-6, insulin and prostaglandin E2, and has been associated with an increased ER-positive breast cancer risk in obese postmenopausal women [2,8]. The primary tumors in PyMT mice are initially ER-positive, but later, as they transition to the metastatic phenotype, there is loss of ER expression [23,35]. The effects of ovariectomy, nonsteroidal antiestrogens, such as tamoxifen, or aromatase inhibitors on the early stages of PyMT carcinogenesis do not appear to have been reported, but would be of considerable interest in the context of breast cancer chemoprevention, as would their potential for suppressing the stimulatory action of obesity/inflammation.

Micrometastases were present in lungs of all the animals in both dietary groups at the end of the experimental period, although with numerically more extensive involvement in HF group. In contrast, in their study of female mice with cosegregation of the obesity QTL and the MMTV-PyMT transgene, Gordon *et al.* [25] observed pulmonary metastases in approximately 42% of each dietary group, despite the high body weight gains in the HF-fed animals. Our mice were 12 weeks old when they had completed 8 weeks on the HF or LF diet and a longer interval may be necessary to demonstrate more clearly a stimulatory effect of the HF/high calorie diet on the further, angiogenesis-supported, growth of micrometastases once they were established at the secondary site. The aggressive nature of the MMTV-PyMT phenotype defined the duration of our study and the rapid growth of the precursor hyperplastic lesions and carcinomas *in situ*, perhaps combined with the fact that the mammary glands were only scanned once a week, may also have been responsible for our failure to observe an effect of the HF diet on tumor latency. Gordon *et al.* [25] reported that in their study, female mice fed the HF diet showed a significantly shorter latent period, although this only amounted to a 3-day difference from that of the LF-fed animals.

Lin *et al.* [23] found that as the MMTV-PyMT tumors underwent early carcinomatous transition there was infiltration by macrophages and increased vascularization in the peritumoral adipocyte-rich stromal tissue; later, with progression to a more advanced stage, the tumors were associated with a reactive stroma consisting of fibroblasts and inflammatory cells in an eosinophilic matrix. Similar cellular changes with associated angiogenesis occurred in the present study and were enhanced by feeding the HF diet. The same characteristics were observed by Wagner *et al.* [36] in the peritumoral adipose tissue of a mouse melanoma model and were associated by overexpression of VEGF, MCP-1, and IL-6.

Although in most situations, VEGF is the principal angiogenic factor, several of the cytokines and related proteins which are regarded as biomarkers of chronic inflammation also promote neovascularization; prominent among these are leptin, TNF-α and MCP-1 [15,37], all three of which are upregulated in obesity [15,38]. Elevated plasma leptin concentrations in mice fed a HF diet have been reported previously [39], and the circulating levels of leptin and MCP-1 were elevated in our HF group of tumor-bearing mice, but there was no increase in the plasma TNF-α, perhaps because this proinflammmatory cytokine acts primarily via a paracrine mechanism, as suggested by the finding by Shah *et al.* [40] that while the plasma levels were unchanged there was an increase in adipose tissue TNF-α mRNA expression in HF-fed mice. We did not assay VEGF levels in our study, but it has been found that myeloid cells, including macrophages, promote angiogenesis by a VEGF-independent mechanism [41]. Recent studies have shown that adipocytes from obese adipose tissue, macrophages, and mammary tumor cells, when cultured alone *in vitro* or when interacting together, express high levels of proangiogenic MCP-1 and VEGF [42].

## 5. Concluding Remarks and Future Directions

Our study is the first to show that MCP-1 was increased in visceral adipose, mammary adipose tissue and in the tumors of non-immunodeficient mice fed a high-fat diet, indicating a direct complication of obesity and chronic inflammation associated with breast cancer development and progression. This study compares mammary tumor development/progression in MMTV-PyMT transgenic mice fed either a low fat or very high fat diet from 4–12 weeks of age; a fast progressing model which does go through stages similar to some human breast cancers. The transgenic mouse model is maintained on the FVB/N strain which has been shown in some studies to be resistant to the effects of a high-fat diet resulting in moderate weight gain. Interestingly, we found that “visceral” fat weight was increased to a greater degree than was body weight indicating a body condition that reflects an individual with “metabolically obese-normal weight.” Based on our results, we suggest that an inflammatory state exists in this animal model which is associated with either excess adiposity or high-fat diet consumption to impact mammary cancer progression.

In experimental obesity studies, approaches are needed to limit macrophage infiltration through macrophage ablation and blocking MCP-1 production during tumor development should target the tumor microenvironment. The interactions between adipose tissue and tumor MCP-1 would suggest a mechanism by which diet-induced obesity produces local inflammation and increases MCP-1 production at a paracrine/autocrine level that creates a microenvironment favorable for breast tumor progression.
